# ‘Bouncing Back’ From Subclinical Malaria: Inflammation and Erythrocytosis After Resolution of *P. falciparum* Infection in Gambian Children

**DOI:** 10.3389/fimmu.2022.780525

**Published:** 2022-01-28

**Authors:** Jason P. Mooney, Sophia M. DonVito, Maimuna Jahateh, Haddy Bittaye, Marianne Keith, Lauren J. Galloway, Mortala Ndow, Aubrey J. Cunnington, Umberto D’Alessandro, Christian Bottomley, Eleanor M. Riley

**Affiliations:** ^1^ Institute of Immunology and Infection Research, School of Biological Sciences, University of Edinburgh, Edinburgh, United Kingdom; ^2^ Medical Research Council Unit in The Gambia at the London School of Hygiene and Tropical Medicine, Fajara, Gambia; ^3^ Institute of Infection, Immunity and Inflammation, University of Glasgow, Glasgow, United Kingdom; ^4^ Section of Paediatric Infectious Disease, Department of Infectious Disease, Imperial College London, London, United Kingdom; ^5^ Department of Infectious Disease Epidemiology, London School of Hygiene and Tropical Medicine, London, United Kingdom

**Keywords:** malaria, *Plasmodium*, subclinical, asymptomatic, erythrocytosis, inflammation, falciparum, Gambia

## Abstract

Recent malaria is associated with an increased risk of systemic bacterial infection. The aetiology of this association is unclear but malaria-related haemolysis may be one contributory factor. To characterise the physiological consequences of persistent and recently resolved malaria infections and associated haemolysis, 1650 healthy Gambian children aged 8–15 years were screened for *P. falciparum* infection (by 18sRNA PCR) and/or anaemia (by haematocrit) at the end of the annual malaria transmission season (t_1_). *P. falciparum*-infected children and children with moderate or severe anaemia (haemoglobin concentration < 11g/dl) were age matched to healthy, uninfected, non-anaemic controls and screened again 2 months later (t_2_). Persistently infected children (PCR positive at t_1_ and t_2_) had stable parasite burdens and did not differ significantly haematologically or in terms of proinflammatory markers from healthy, uninfected children. However, among persistently infected children, IL-10 concentrations were positively correlated with parasite density suggesting a tolerogenic response to persistent infection. By contrast, children who naturally resolved their infections (positive at t_1_ and negative at t_2_) exhibited mild erythrocytosis and concentrations of pro-inflammatory markers were raised compared to other groups of children. These findings shed light on a ‘resetting’ and potential overshoot of the homeostatic haematological response following resolution of malaria infection. Interestingly, the majority of parameters tested were highly heterogeneous in uninfected children, suggesting that some may be harbouring cryptic malaria or other infections.

## Introduction

In addition to an estimated 229 million clinical cases of malaria globally in 2019 (1), there is a large, hidden pool of *Plasmodium* spp. infections that go undiagnosed due to the absence of fever or other characteristic clinical signs (2). Many of these infections are below the limit of detection of standard diagnostics and may be only intermittently detectable by highly sensitive PCR (3) due to sequestration in deep tissues, including the spleen (4). There is considerable debate as to the health and developmental consequences of subclinical *Plasmodium* spp. infections, particularly in children, as well as their role in the acquisition of sustained antimalarial immunity and their contribution to malaria transmission (2, 5). Moreover, as these asymptomatically infected individuals rarely seek antimalarial drug therapy, infections may persist for months or years (6) and seed continual infection of mosquitoes in areas of highly seasonal transmission, maintaining parasite circulation across dry seasons.

One potential consequence of persistent, asymptomatic malaria infection is chronic, low grade, parasite-driven inflammation that may in turn lead to disturbed immune homeostasis and increased susceptibility to other infections or immune disorders. Specifically, individuals with recent or low-density malaria infections are at increased risk of invasive bacterial disease caused, primarily, by enterobacteriaceae (7). In a pilot study of asymptomatically infected children in Burkina Faso, we observed evidence of persistent haemolysis together with raised plasma haem and haem oxygenase 1 (HO-1) (8), features previously associated with neutrophil dysfunction in children (9) and an inability to control non-Typhoidal *Salmonella* infections in mice (10). In this cohort, plasma concentrations of the anti-inflammatory cytokine IL-10, which can directly activate HO-1, were also raised in persistently infected individuals compared to uninfected controls (8).

In this study, we sought to characterise systemic markers of anaemia, haemolysis and inflammation in children with persistent or recent asymptomatic *Plasmodium falciparum* infection, or anaemia, living in a low-transmission environment in The Gambia.

## Materials and Methods

### Study Design and Sample Collection

**Figure 1:**
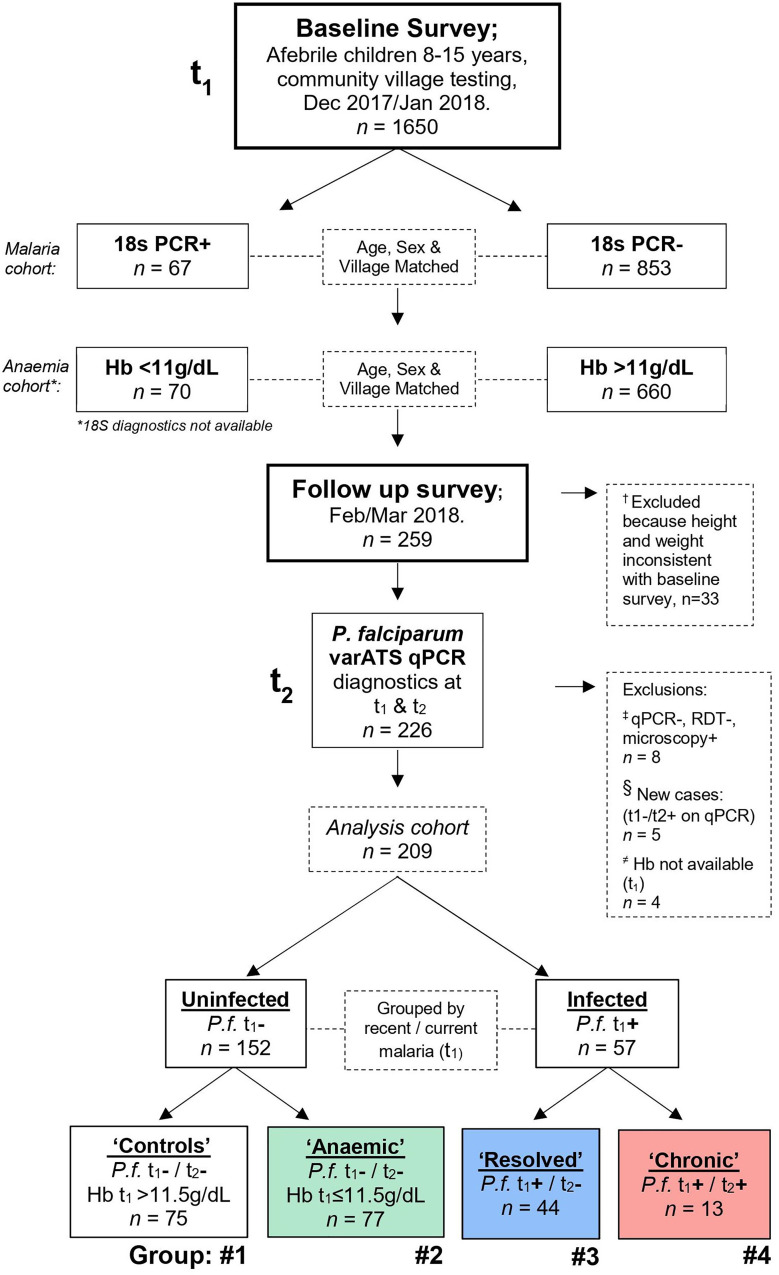
Study design. A baseline cross-sectional field survey was conducted during the beginning of the dry season, from December 2017 to January 2018 (time point 1, t_1_) in the Upper River Region of The Gambia. 1650 children were recruited, and blood sampled by finger prick. Based on initial diagnostics completed at the time [*P. falciparum* 18S PCR or haemoglobin (Hb)], 333 children were identified for recall in the follow up survey. This broad group included all identified *P. falciparum* positive children and those with anaemia (Hb <11 g/dL); along with age-, sex- and village-matched controls in a 1:1 ratio or where sufficient controls where available a 1:2 ratio. Specifically, 67 PCR+ and 126 PCR- children were identified from the first 920 children sampled, and 70 anaemic children and 140 non-anaemic controls identified from the remaining pool of 730 children. Of these 333 children recalled, 259 accepted transport to Basse regional hospital between February and March 2018 (time point 2, t_2_) and blood was collected by venepuncture. Upon comparing changes in patient height and weight between t_1_ and t_2_, 33 children were excluded because they may not have participated in the baseline survey; 14 who decreased in height by more than 5cm and weight by more than 15%, 12 who increased in height by more than 10cm and weight by more than 20%, and 7 with no height and weight data available for comparison (†; see *Materials and Methods* and **Table S3**). Retrospectively, qPCR diagnostics were repeated for *P.f.* varATS and children were first grouped by recent malaria infection at t_1_. Of the uninfected at t_1_ (*n*=156), children were further grouped by Hb concentration; non-anaemic (*n*=75) and anaemic (*n*=77), (≠) with 4 children excluded due to no Hb reading available. Of the children infected at t_1_ (*n*=57), 13 remained persistently infected and 44 had resolved their infection at recall (t_2_). Children who were negative by qPCR and RDT, but positive by microscopy in t_1_ were excluded (‡; **Table S4**). (§) Due to malaria transmission in The Gambia being highly seasonal, the five children with acquired infections during the study period (‘New’) were excluded from further analysis, as these were unlikely to be locally acquired. Thus, the four groups were: #1 Uninfected ‘Controls’, #2 Uninfected ‘Anaemic’ controls, #3 ‘Resolved’ (those who were positive at t_1_ and negative by t_2_) and #4 ‘Chronic’ (those who maintained infections throughout both study time points).

At the end of the malaria transmission season in December 2017/January 2018 (t_1_), a cross-sectional survey of children aged 8–15 years, residing in twenty-nine villages in the Upper River Region of The Gambia, was conducted to identify 1650 children in good general health and with no evidence of fever (body temperature <38°C) for inclusion in the study (**Figure 1**). Additional exclusion criteria included participation in another ongoing research study; any signs of significant ill health (e.g. cardiovascular, pulmonary, renal, hepatic, neurological, dermatological, endocrine, malignant, infectious, immunodeficiency, psychiatric and other disorders); other known medical conditions (e.g., HIV infection, sickle cell disease or thalassaemia); recent antimalarial or antibiotic treatment (within the previous month). Height, weight, sex, age, and village of residence were recorded. Finger prick blood samples were obtained for malaria microscopy (Giemsa stained thick films), rapid diagnosis by lateral flow assay for *P. falciparum* histidine-rich protein II (*Pf*HRP2) (SD BIOLINE Malaria Ag *P.f*, Abbott), preparation of dried blood spots for *P. falciparum* qPCR analysis (see below) and haemoglobin (Hb) estimation by Hemocue (Hb201+, Radiometer). In a follow up survey conducted in February/March 2018 (t_2_), children identified as parasite positive by 18S PCR (n = 67) in the baseline survey were age, sex, and village matched to children with no detectable parasitaemia; anaemic children (Hb < 11 g/dL, n = 70) were similarly matched to children with Hb ≥11 g/dL, respectively. These children were invited for a second clinical examination and blood sample collection at Basse Regional Hospital. The study was approved by The Medical Research Council Gambia (MRCG) Scientific Coordinating Committee and by the Gambia Government/MRCG Joint Ethics Committee (reference 1545). Prior to enrolment, verbal assent was obtained from study participants and verbal or written consent was obtained from their parent or guardian. Stored plasma samples from 12 Gambian children with acute clinical malaria (11, 12) (**Table S1**) were used as comparators in some assays.

### *P. falciparum* Diagnostic PCR

*P. falciparum* diagnostic PCR was performed in two stages. For screening (t_1_), PCR for 18S ribosomal RNA was performed as described previously (13, 14). For definitive diagnosis in the final cohort of children seen at both t_1_ and t_2_, qPCR against the *var* gene acidic terminal sequence (*var*ATS) of *P. falciparum* was performed as described previously (15). Briefly, DNA from dried blood spots was extracted using the QIAamp 96 DNA QIAcube HT Kit (Qiagen). For *var*ATS qPCR, samples were run in duplicate against a universal standard [NIBSC code 04/176 (16)]. Samples were deemed positive for *P. falciparum* DNA if both replicates were detectable at Ct<40 cycles. Discrepant samples (where only 1 of the 2 replicates were detected at Ct<40) were run again in duplicate; only samples which were positive in both replicates on the same plate were deemed infected. Reaction parameters for both 18S and *var*ATS qPCR are described in the supplemental methods.

### Blood Sample Preparation

In the follow up survey (t_2_), approx. 10 mL venous blood was collected into EDTA vacutainers (BD). Complete blood counts were performed using an automated haematology analyser (M series, Medonic). The remaining whole blood was layered onto Ficoll (Histopaque^®^-1077; Hypaque) and centrifuged at 500 x *g* for 30 minutes (brake off). Plasma was removed and stored at -80°C. Cell pellets were cryopreserved in liquid nitrogen for future studies.

### Plasma Analysis

Enzyme-linked immunosorbent assays (ELISAs) were conducted according to manufacturers’ instructions to measure plasma concentrations of haemopexin (OKIA00066, Aviva Systems Biology) and erythropoietin (EPO, DY286-05, R&D Systems), at a dilution of 1:40,000 or undiluted, respectively. Colorimetric determination of haem in undiluted plasma samples was conducted according to manufacturers’ instructions (MAK316-1KT, Sigma-Aldrich). Luminex microbead-based suspension array (LXSAHM, R&D Systems) was used according to manufacturer instructions to detect plasma concentrations of IL-10, CD163, IFN-γ, IL-6, TNF-α, CXCL10, G-CSF, C5a, and S100a9 at plasma dilutions of 1:2. Finally, for detection of ferritin, transferrin, C reactive protein (CRP), LPS binding protein (LBP), myeloperoxidase (MPO), and matrix metallopeptidase 9 (MMP-9) by Luminex, plasma was diluted 1:100. Plasma protein concentrations were determined from standard curves after subtraction of background values, calculated using MS Excel. The upper and lower limits of quantification (ULOQ and LLOQ, respectively), and manufacturers’ codes for each analyte, are reported in **Table S2**.

### Data Management and Statistical Analysis

Field data were collected and stored on portable electronic devices using REDcap data management software (17). Electronic data were then exported to MS Excel for analysis. Comparisons between uninfected children and those with resolved or chronic *P. falciparum* parasitaemia were performed using Kruskal-Wallis/Dunn’s test with a Bonferroni adjustment for multiple testing. Correlations were assessed using Pearson’s correlation coefficient. The null hypothesis of zero correlation was tested using a Wald test for sample sizes >20 and a permutation test for sample sizes ≤20. To mitigate against the impact of potential outliers, the ROUT outlier test was applied to individual data points of concern. Percentages were compared using the chi-square test. All statistical analyses were performed using GraphPad Prism (v.9.1.0) or in R Studio (v4.0.4). A *p* value of <0.05 was considered statistically significant.

## Results

### Cohort Characteristics

Of the 1650 healthy, afebrile children recruited at t_1_, 920 were screened by 18S PCR and 67 were positive (“infected”). Of the remaining 730 children, who were not screened by 18S PCR at t_1_, 70 had an Hb concentration <11g/dL (“anaemic”) **(** **Figure 1** **)**. As far as possible, these children were age (+/-1 year), sex and village matched to children who were either qPCR negative or who had a Hb ≥11 g/dL, respectively. This generated a cohort of 333 children who were recalled at t_2_, of whom 259 attended for examination. Children whose height declined by >5 cm together with a weight decline of >15% (*n* = 14), or whose height increased by >10cm together with a weight increase of >20% (*n* = 12), or for whom reliable data on height and weight were not available (*n* = 7), could not be confirmed as being the same child and were excluded from further study (**Table S3**).

Of the 226 children seen at t_1_ and t_2_, the status of 5 children who were qPCR negative at t_1_ but qPCR positive at t_2_ was deemed uncertain and they were omitted from the analysis, particularly as these may have represented new infections acquired during travel beyond the local area (as transmission is highly seasonal). Furthermore, 8 children who were microscopy positive at t_1_ but negative by both qPCR and RDT, and 4 children for whom Hb concentration was not available at t_1_, were also omitted (**Table S4**) leaving a final t_2_ cohort of 209 children (**Figure 1**). Of these 209 children, 13 were *var*ATS qPCR positive for *P. falciparum* at t_1_ and t_2_ (deemed “chronically infected”) and 44 were positive at t_1_ but negative at t_2_ (deemed “resolved” infections). Of the 152 uninfected children, 77 had an [Hb ≤11.5 g/dL, defined as anaemia in children (18)] at t_1_ and were deemed “anaemic”, leaving 75 who were neither infected nor anaemic (healthy controls).

**Figure 2:**
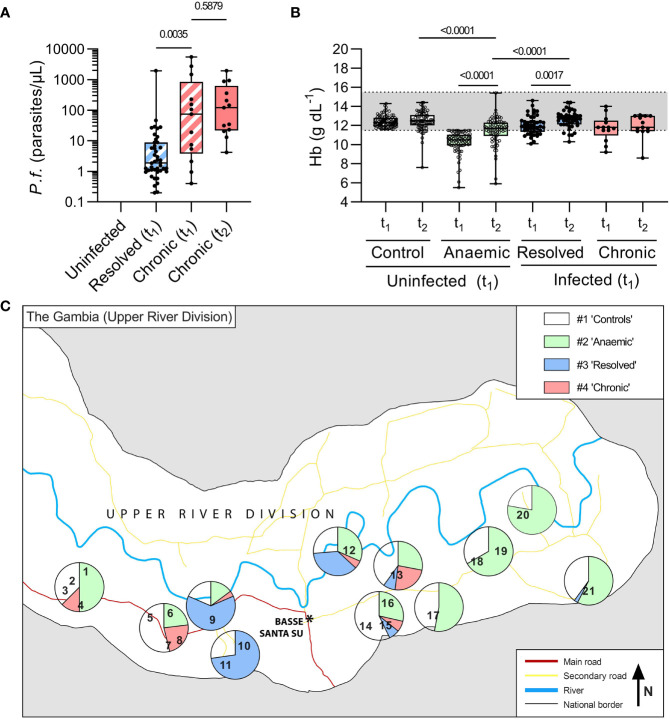
Parasitaemia and anaemia in uninfected children and children with resolved or persistent *P. falciparum* infections in the Upper River Division of The Gambia. In total, 209 age, sex, and village-matched children were followed over the dry season from initial recruitment in the baseline survey (t_1_) to follow up (t_2_) and grouped based on presence of *P. falciparum* DNA by varATS qPCR at both time-points. ‘Chronic’ children were qPCR positive at both t_1_ and t_2_ (*n*=13, 6%), ‘resolved’ (*n*=44, 21%) were PCR positive at t_1_ and negative at t_2_, and the remaining (*n*=156, 73%) children were ‘Uninfected’. The uninfected children were defined as anaemic or not based on Hb concentration at t_1_ (Hb ≤ 11.5g/dL, *n*=77). Of those who were qPCR positive at t_1_ (*n*=57), 77% resolved their infection by t_2_. **(A)** Parasite burden, determined by varATS qPCR, in parasites/μL. Mann Whitney test used for comparison between Resolved (t_1_) and Chronic (t_1_), hatched boxes. Wilcoxon matched-pairs signed rank test used for comparison between Chronic (t_1_) and Chronic (t_2_), with *p*-values shown. **(B)** Haemoglobin (Hb g/dL) by hemocue at baseline (t_1_) and recall (t_2_). **(C)** The study participants came from 21 villages (numbered, see **Table S6**). The proportions of children in each group in each village are represented by pie charts. Where only very small numbers of children from a village were tested, villages have been clustered for analysis, with a breakdown per village/cluster reported in **Table S6**.

*P. falciparum* infections (both chronic and resolved) were more prevalent in the central and western part of the study area than in the eastern part (**Figure 2**). However, the overall rate of subclinical parasitaemia at t_1_ (~9%) was lower than the ~14% anticipated from previous surveys (19), and a high proportion of infections (77%) resolved in the approximately 2 months between t_1_ and t_2_, leaving the study underpowered for some analyses of persistent infections.

The median recall time (i.e., time between t_1_ and t_2_) was 64 days (IQR 62-66) and did not differ significantly among the groups. There were no significant differences between the groups in age or sex although uninfected, anaemic children were shorter and weighed less than non-anaemic controls (**Table 1**). Parasitaemia at t_1_ was significantly higher among children whose infections persisted at t_2_ than among children whose infections had resolved at t_2_ (median 74 parasites/µL vs. 2 parasites/µL; *p* = 0.003). Among children with persistent chronic infections, median parasitaemia did not differ significantly between t_1_ and t_2_ (74 parasites/µL and 120 parasites/µL, respectively; *p* = 0.59) (**Figure 2A**). Haemoglobin concentrations did not differ significantly between t_1_ and t_2_ for either healthy controls or persistently infected children, but haemoglobin concentrations increased significantly between t_1_ and t_2_ among uninfected anaemic children whilst remaining significantly lower than among healthy controls (median = 11.7 g/dL (IQR 11-12.3) vs. 12.2 g/dL (11.5-12.7), respectively; *p* < 0.0001) and than among those whose infections resolved (**Figure 2B**). Mean haemoglobin concentrations of persistently infected children were at the bottom of the normal range at t_1_ and t_2_ but small numbers precluded the drawing of any substantial conclusions.

**Table 1:** Demographics of the study population.

Characteristic	#1 ‘Control’(*n*=75)	#2 ‘Anaemic’(*n*=77)	#3 ‘Resolved’(*n*=44)	#4 ‘Chronic’(*n*=13)	*p* value
Age (years)	11 (9-12)	10 (9-12)	11 (10-12)	11 (10-12)	0.14
Sex (# Female, %)	35 (47%)	28 (36%)	12 (27%)	6 46%)	0.18
Time between baseline and follow up (days)	64 (61-67)	64 (63-67)	63 (62-64)	64 (57-79)	0.35
Weight at follow up (kg)	31 (27-38)	28 (25-31)	29 (24-35)	31 (25-36)	0.04^‡^
Height at follow up (m)	1.4 (1.3-1.5)	1.4 (1.3-1.4)	1.4 (1.3-1.4)	1.4 (1.4-1.5)	0.04^‡^
Temperature at follow up (°C)	36.9 (36.4-37.1)	36.6 (36.2-37)	36.8 (36.5-37)	36.8 (36.5-37.1)	0.26
% change in weight	3 (0-5)	2 (-1-4)	1 (-1-3)	2 (-2-5)	0.08
change in height (cm)	1 (1-2)	2 (1-3)	1.5 (1-2)	2 (0-4)	0.006
Parasitaemia at baseline (parasites/µL)	–	–	2 (1-7)	74 (6-228)	0.003^†^
Parasitaemia at follow up (parasites/µL)	–	–	–	120 (23-358)	–

Median values with interquartile range (IQR, Q1 and Q3) in brackets, unless otherwise noted for sex values. p values between groups calculated using a Kruskal-Wallis rank sum test, ^†^ or Mann-Whitney test baseline parasitaemia. ^‡^Between uninfected controls (group #1) and uninfected anaemic children (group #2), after Dunn’s multiple comparisons test, p = 0.02 for weight and p = 0.05 for height.

### Haematology

**Figure 3:**
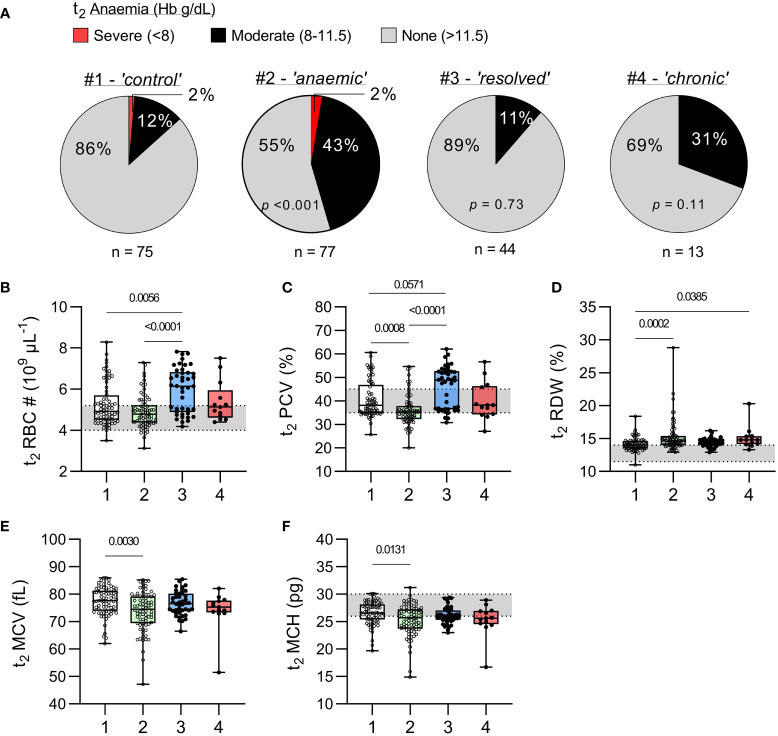
Haematological parameters in uninfected children and children with resolved or persistent *P. falciparum* infections. **(A)** Prevalence of anaemia at t_2_, as defined by WHO (18), for each study group and p-value from a chi-square test comparing with the uninfected group (χ^2^). **(B–F)** Complete blood counts were performed on venous blood from t_2_ for erythrocyte parameters; **(B)** total red blood cell (RBC) number (#), **(C)** packed cell volume (PCV) or haematocrit (%), **(D)** red cell distribution width (RDW, %), **(E)** mean cell volume (MCV, fL), and **(F)** mean cell haemoglobin (MCH, pg). Grey shaded areas represent normal paediatric reference ranges (20). Data shown as box plots with min/max whiskers where dots represent each participant. Significant *p* values shown, calculated using a Kruskal-Wallis rank sum test followed by a *post-hoc* Dunn’s test with Bonferroni adjustment for multiple comparisons. Group IDs: #1 ‘Controls’, #2 ‘Anaemic’, #3 ‘Resolved’, and #4 ‘Chronic’.

The prevalence of moderate anaemia [Hb between 8 and 11.5 g/dL (18)] was similar among uninfected children (12%) and children with resolved infections (11%, *p* = 0.73), and similar to that in children with chronic infections (31%, *p* = 0.11) (**Figure 3**). Severe anaemia (Hb ≤ 8 g/dL) was observed only among uninfected children, although this may be a chance finding given the much larger number of children in this group. Somewhat surprisingly, haematocrit (packed cell volume; PCV) and red blood cell (RBC) counts were significantly higher [and above the normal paediatric range (20)] among children with recently resolved malaria infections when compared with children with no evidence of recent infection, suggesting a rapid rebound in erythropoiesis once their malaria infections resolved. Although PCV and RBC counts did not differ significantly between children with persistent infections and the other groups of children, the power of these comparisons is limited by the small number of chronically infected children.

Interestingly, haematological indicators of uninfected children who were anaemic at t_1_ improved somewhat by t_2_ with median Hb being significantly higher (p < 0.001) (**Figure 2B**) and median RBC count being within the normal range (**Figure 3B**). Nevertheless, the uninfected anaemic group showed evidence of persisting in red cell abnormalities, with significantly increased red cell width, reduced PCV, reduced mean cell volume and reduced mean cell haemoglobin concentration when compared to healthy non-anaemic controls (**Figures 3C–F**).

**Figure 4:**
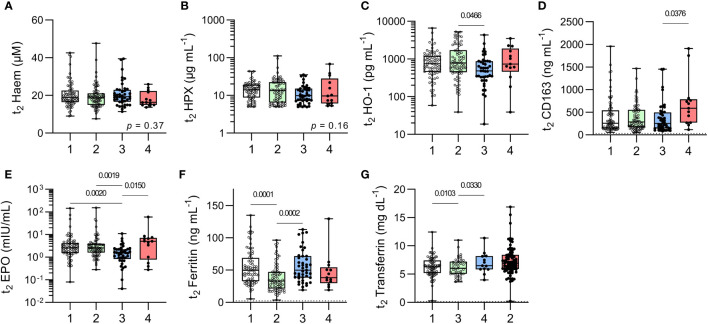
Markers of erythropoiesis in uninfected children and children with resolved or persistent *P. falciparum* infections. Concentrations of soluble proteins in plasma for **(A)** Haem, **(B)** Haemopexin (HPX), **(C)** haemoxygenase-1 (HO-1), **(D)** soluble CD163, **(E)** erythropoietin (EPO), **(F)** Ferritin (iron load), and **(G)** Transferrin (ferric-ion delivery). Data shown as box plots with min/max whiskers where dots represent each participant. Significant *p* values shown, calculated using a Kruskal-Wallis rank sum test followed by a *post-hoc* Dunn’s test with Bonferroni adjustment for multiple comparisons. Group IDs: #1 ‘Controls’, #2 ‘Anaemic’, #3 ‘Resolved’, and #4 ‘Chronic’.

To further explore haematological responses to subclinical malaria infections, plasma concentrations of haem, haemopexin, HO-1 and soluble CD163 (the high affinity scavenger for haptoglobin-haemoglobin complexes), all of which are markers of haemolysis (**Figures 4A–D**) and of erythropoietin (EPO), ferritin, transferrin (markers of iron status and mobilisation) (**Figures 4E–G**) were measured at t_2_. In contrast to previous observations (8), the only evidence of ongoing hemolysis in the persistently infected children was a raised median concentration of CD163 (**Figure 4D**) and their iron status markers were within the normal range. Children with recently resolved malaria infections had HO-1 concentrations that were somewhat lower than the other children (**Figure 4C**) and median EPO concentrations that were significantly lower when compared to all other groups (**Figure 4E**). Meanwhile, uninfected, anaemic children had significantly lower ferritin concentrations and significantly higher transferrin concentrations when compared to healthy uninfected children (**Figures 4F, G** **)** suggesting that their anaemia may be due, in part, to iron insufficiency.

### Immunological and Inflammatory Responses

**Figure 5:**
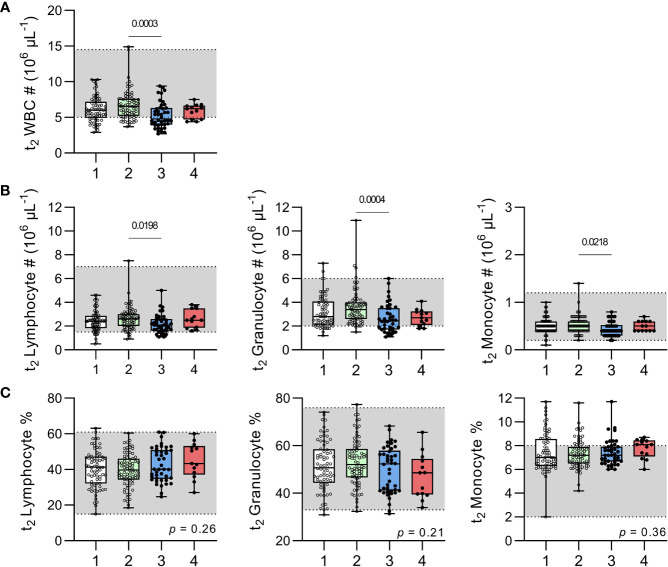
Leucocyte numbers and proportions in uninfected children and children with resolved or persistent *P. falciparum* infections. Complete blood counts were performed on venous blood for **(A)** total white blood cell (WBC) numbers (#). Subpopulations of lymphocyte, granulocyte and monocytes enumerated and shown as total number **(B)** or percentage **(C)**. Grey shadow boxes represent normal paediatric reference ranges (20). Data shown as box plots with min/max whiskers where dots represent each participant. Significant *p* values shown, calculated using a Kruskal-Wallis rank sum test followed by a *post-hoc* Dunn’s test with Bonferroni adjustment for multiple comparisons. Group IDs: #1 ‘Controls’, #2 ‘Anaemic’, #3 ‘Resolved’, and #4 ‘Chronic’.

Overall, leucocyte counts fell within the normal paediatric range for the majority of children at t_2_ (**Figure 5**) with the only exception being that the median total leucocyte count of children with recently resolved malaria infections (4.8 x10^6^/µL; IQR 4-6.3) was slightly below the normal range of 5-14.5 x10^6^/µL (20). Children with recently resolved malaria infections also had significantly lower total white blood cell counts and lower granulocyte, monocyte and lymphocyte counts than the uninfected, anaemic group (**Figure 5**). However, median leucocyte proportions concentrations did not differ significantly among the groups and were within the normal range (**Figure 5C**).

**Figure 6:**
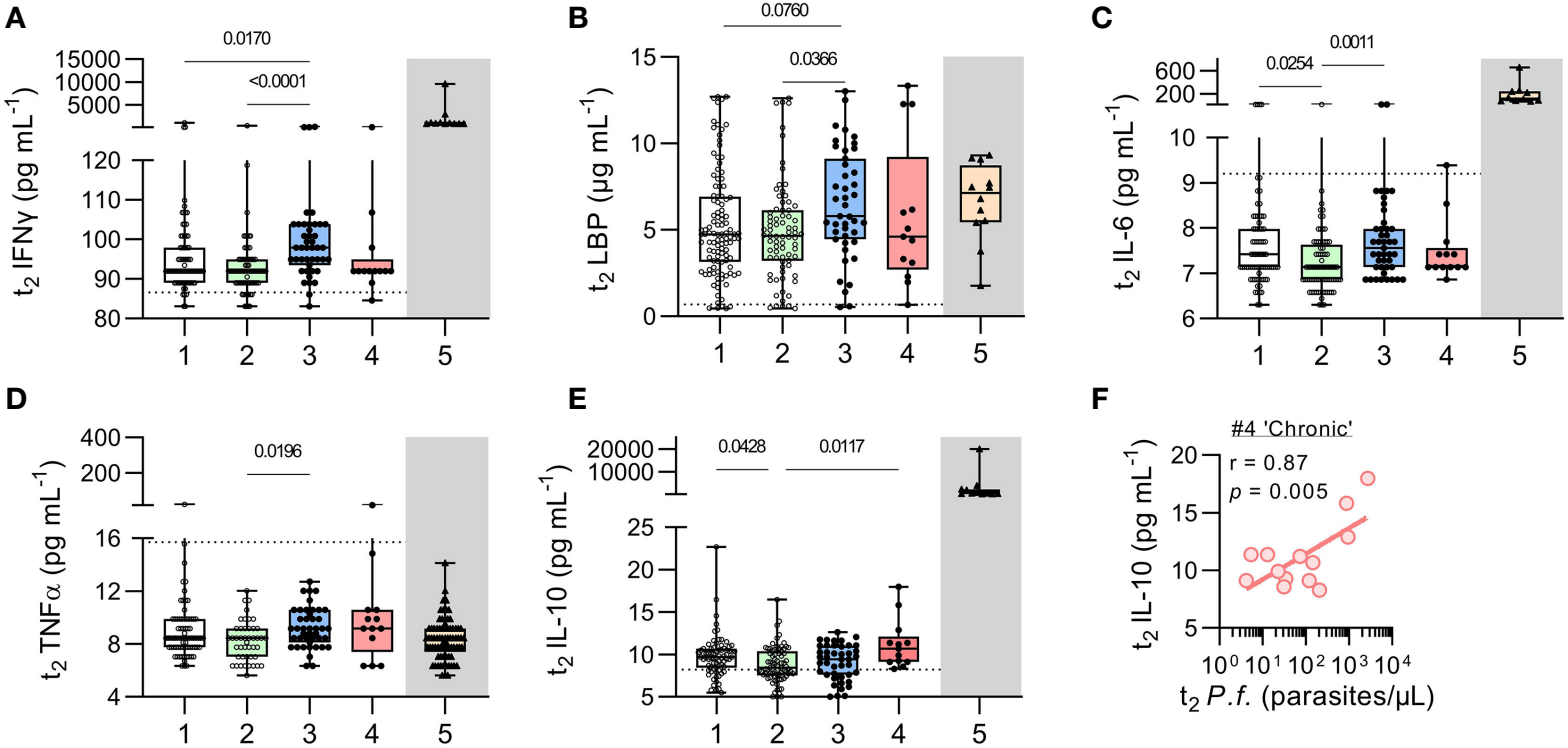
Inflammatory cytokines in uninfected children and children with resolved or persistent *P. falciparum* infections. Concentrations of soluble proteins in plasma measured by Luminex multiplex bead-based assay (Invitrogen) for **(A)** IFNγ, and **(B)** LPS binding protein (LBP), **(C)** IL-6, **(D)** TNFα, and **(E)** IL-10. As a reference point (grey box), protein concentrations for 12 Gambian children with acute, clinical malaria (11, 12) are shown (with patient demographics in **Table S1**). Dotted lines represent the ‘lower limit of quantification’ (LLOQ, **Table S2**). Data shown as box plots with min/max whiskers where dots represent each participant. Significant *p* values shown, calculated using a Kruskal-Wallis rank sum test followed by a *post-hoc* Dunn’s test with Bonferroni adjustment for multiple comparisons. **(F)** Given the increase in IL-10 observed in children with chronic malaria parasite infection, correlation analysis of current (t_2_) parasite infection with plasma IL-10 was performed. Pearson’s correlation (r) shown with fitted regression line and *p* value from a permutation test. Group IDs: #1 ‘controls’, #2 ‘anaemic’, #3 ‘resolved’, #4 ‘chronic’, & #5 ‘clinical malaria’.

Systemic inflammation is a feature of symptomatic malaria infections (21); acquired immunity is associated with the ability to modulate this inflammation (21) such that raised concentrations of circulating anti-inflammatory cytokines – especially IL-10 – may be the only sign of ongoing immune disturbance (8, 22, 23). However, systemic inflammation has also been observed in subclinical, asymptomatic infections (24–26). We therefore measured plasma concentrations of a range of cytokines and inflammatory markers including IFN-γ, LPS binding protein (LBP), IL-6, TNF-α, and IL-10 (**Figure 6** and **Figure S1**).

Concentrations of pro-inflammatory markers did not differ significantly between persistently infected and uninfected children (**Figures 6A–D**) and were noticeably lower than in a historical cohort of children with acute, symptomatic malaria infection (Group 5). However, when compared to uninfected children, children with recently resolved malaria infections had modestly but significantly higher concentrations of IFN-γ and LBP (**Figures 6A, B** **)** and their concentrations of IL-6 and TNFα were significantly higher than among uninfected anaemic children (**Figures 6C, D** **)**. No significant differences were observed between any of the groups in concentrations of CRP, C5a, S100a9, MPO, CXCL10, G-CSF or MMP-9 (**Figure S1**). By contrast, IL-10 concentrations were modestly raised (albeit at much lower levels than in acutely infected symptomatic children) among the persistently infected children (**Figure 6E**). Moreover, in these chronically infected children, IL-10 concentrations were highly correlated with parasite density (r = 0.87, *p* = 0.005) (**Figure 6F**).

It was noticeable, however, that concentrations of pro-inflammatory markers varied considerably within the groups, including in apparently uninfected children. Indeed, concentrations of inflammatory proteins in some children in every group were as high as, or higher than, those of acutely malaria-infected children (**Table S7**). For example, 22% of children without any evidence of malaria infection had C-reactive protein (CRP) concentrations above the normal threshold of 1mg/L (20) with values up to 3.9 mg/L (**Figure S2**). Although these CRP concentrations are lower than those seen in children with acute clinical malaria (median 7.1, IQR 5.5 - 8.0), they may be indicative of a low level inflammatory state. Together, these data suggest that many apparently healthy Gambian children may be living with persistent, low-grade inflammation that may be indicative of cryptic infection (with malaria, or other co-endemic pathogens).

## Discussion

After decades of control activities, the Upper River Region of The Gambia is now classified as an area of low, seasonal malaria transmission with significant spatial heterogeneity and year-to-year variation (27, 28). Malaria control activities have been focused on traditional ‘at risk’ groups (children under 5 and women of childbearing age) and this seems to have led in recent years to a shift in the burden of malaria parasite infection to somewhat older children (29). These infections are frequently very low density (detected by PCR or by rapid diagnostic tests for malaria antigens in individuals who are parasite negative by microscopy) and tend to be subclinical. Such infections are, however, associated with increased likelihood of anaemia (29) and risk of invasive bacterial disease (7, 30).

In studies in mice, malaria-induced haemolysis and induction of HO-1 lead to neutrophil dysfunction and increased susceptibility to invasive non-Typhoidal *Salmonella* (iNTS) infections (10, 31). Similarly, in children with acute (symptomatic) malaria infections, prolonged neutrophil dysfunction is associated with haemolysis and HO-1 induction (9). Importantly, in a pilot study in Burkinabé children, we found evidence that subclinical *P. falciparum* infections also induce ongoing haemolysis, with raised concentrations of plasma haem and HO-1 (8). Furthermore, in a study of adult sepsis patients in the UK, upregulation of both HO-1 and IL-10 were strongly associated with disease severity, and IL-10 concentration was predictive of HO-1 concentration (32). Given that subclinical malaria can cause haemolysis (8, 33), that haemolysis induces HO-1 (34), and that HO-1 negatively affects neutrophil function (10) and mediates many of the anti-inflammatory effects of IL-10 (35), this present study was designed to further investigate the haematological and immunological impacts of subclinical malaria parasite infections and their resolution.

Parasite prevalence at the end of the annual rainy season (t_1_) in this cohort was approx. 9%, somewhat lower than in recent surveys in the same area at the same time of year where prevalence ranged from 13-31% (27, 28, 36). In addition, the proportion of the infections persisting for two months during the subsequent dry (non-transmission) season (23%) was lower than predicted from our previous pilot study in Burkina Faso (95% persistent for 35 days) (8) leaving the study underpowered for some of the subsequent analyses.

Among children with persistent infections, parasite density did not change significantly over the two months of follow up. This is consistent with data from Burkina Faso (8), as are our findings that the prevalence of mild to moderate anaemia did not differ between children with persistent subclinical infections and uninfected control children, and that persistently infected children had raised levels of circulating IL-10. Prah et al. also found no significant differences in haemoglobin concentrations between uninfected children and those with asymptomatic infections in Ghana (37).

However, in contrast to our previous study (8), the only obvious abnormalities in markers of haemolysis, iron handling and inflammation in the persistently infected group were modestly, but significantly, higher concentrations of CD163 and IL-10. CD163 is the high affinity scavenger receptor for the haemoglobin-haptoglobin complexes and is shed into plasma by activated monocytes (38). CD163 is one of a suite of proteins in the haemoglobin degradation and iron recycling pathways, including HO-1 (35), and its expression is upregulated by IL-10 (39). The modest increase in CD163, together with the modestly raised IL-10 concentrations, suggests that IL-10, rather than ongoing haemolysis, may be the regulator of CD163 in this cohort of persistently infected children. However, the impact of persistent malaria infection on monocyte activation status, inducing membrane bound CD163 and iron recycling, has yet to be fully characterised.

Whilst the lack of marked haematological or immunological disturbances in this cohort of persistently infected children might be interpreted as evidence that subclinical infections are of little physiological consequence, this could be misleading. Firstly, our group of persistently infected children was small and variances within the group were large, providing limited statistical power to detect significant differences. Secondly, parasite densities in the chronically infected children in this study were substantially lower than were seen in Burkina Faso (8). Lastly, the group of supposedly uninfected children was also highly heterogeneous with some children having levels of markers of haemolysis and inflammation that were well outside the reported healthy ranges, indicating that apparently healthy, parasite-free children in malaria endemic areas have ongoing, low-level haemolysis and inflammation.

It is difficult to completely exclude the possibility of current or very recent malaria infection in children living in endemic areas. The presence of PCR detectable parasites may vary from day to day over weeks or months of follow up in individual children (40, 41). Children can maintain the same (genetically identical) parasite infection for many months with parasites only being intermittently detectable by microscopy and PCR (41, 42) and the density of subclinical infections can vary through time with windows of undetectable parasitaemia (41). Moreover, a recent study has revealed a hidden biomass of parasites in the spleens of patients undergoing splenectomy, including some patients in whom no circulating parasites could be detected (43). This raises the distinct possibility that our so-called “uninfected” group may actually include children with ongoing infections (malaria or other) and are thus not an entirely appropriate comparator group. Moreover, parasite derived haemozoin (Hz) is potently pro-inflammatory and can persist in tissues long after clearance of malaria infections (44) such that immune homeostasis may only be restored weeks or months after infections are resolved. These limitations may also explain inconsistencies in findings among other recent studies of asymptomatic or subclinical malaria infections (23–26). In future, repeated, more frequent sampling, including antigen detection for recent infection, and/or curative drug treatment to remove any cryptic parasites, will be required to ensure children are genuinely free of malaria parasite infection.

The high proportion of subclinical infections that appeared to spontaneously resolve over the 8 weeks of follow up did, however, give us an opportunity to explore the haematological and immunological consequences of recent infection. Compared with uninfected ‘control’ children, those with ‘resolved’ infections demonstrated mild erythrocytosis, generalised leucopenia and mild but statistically significant systemic inflammation (raised IFN-γ and LBP). Taken together, these observations suggest that clearance of subclinical infections is a mildly inflammatory process – likely dependent upon phagocytosis and degradation of parasitised and uninfected erythrocytes by splenic macrophages (45, 46). Of note, higher LBP concentrations in children with resolved infections may be an indicator of increased intestinal permeability (47) raising the possibility that intestinal damage - due to sequestered parasitised erythrocytes or as a consequence of systemic inflammation - may also underlie the increased susceptibility to invasive bacterial disease in children recovering from malaria infection (7, 31).

Parasite clearance in children with resolving infections may lead to a period of homeostatic erythrocytosis, with a ‘bounce back’ in haematological parameters. In individuals with symptomatic malaria, curative chemotherapy leads to a reversal of bone marrow suppression and accompanying erythrocytosis within 1-2 weeks (48). Importantly, iron handling markers in children with resolving infections did not differ significantly from healthy uninfected controls suggesting that iron availability is not a limiting factor in restoring red cell homeostasis after clearance of asymptomatic malaria infections. This contrasts with our observations of persisting red cell abnormalities in anaemic children without detectable malaria infections, which likely has a different underlying aetiology.

In summary, persisting malaria infections were infrequent in this cohort of Gambian children and the numbers of children with persistent infection were too small for meaningful conclusions to be drawn. However, this study does reveal – for the first time – that resolution of very low density, subclinical *P. falciparum* infection is associated with rapid “bounce back” restoration of red cell homeostasis as well as mild systemic inflammation. The very high levels of LBP observed in some of the study children (whether currently infected with malaria or not) do raise concerns that underlying intestinal inflammation may predispose them to invasive enteric infections. However, the clinical impacts of our observations are difficult to ascertain as subclinical malaria infections are not routinely treated and, in areas of low to moderate malaria transmission, even partially-immune children may oscillate between infected and uninfected status throughout the year. Studies are underway to determine the impact of persistent and resolving infections on neutrophil function and, by extension, susceptibility to coinfections. However, longitudinal studies of malaria infection and secondary bacterial infection in areas of moderate to high endemicity are needed to inform the ongoing debate regarding the risks and benefits of treating subclinical infections (2, 49).

## Data Availability Statement

The raw data supporting the conclusions of this article will be made available by the authors, without undue reservation.

## Ethics Statement

The study was approved by The Medical Research Council Gambia (MRCG) Scientific Coordinating Committee and by the Gambia Government/MRCG Joint Ethics Committee (reference 1545). Prior to enrolment, verbal assent was obtained from study participants and verbal or written consent was obtained from their parent or guardian. Written informed consent to participate in this study was provided by the participants’ legal guardian/next of kin.

## Author Contributions

Study concept and design: JM, UD’A, CB, and ER. Data generation: JM, SD, MJ, HB, MK, LG, MN, and AC. Data analysis: JM, SD, CB, and ER. Statistical review: JM and CB. Drafting and revision of manuscript: JM, SD, CB, and ER. All authors contributed to the article and approved the submitted version.

## Funding

This work was funded by the UK Medical Research Council (MRC) (ER; MR/P000959/2) and the Wellcome Trust (ER; 204804/Z/16/Z).

## Conflict of Interest

The authors declare that the research was conducted in the absence of any commercial or financial relationships that could be construed as a potential conflict of interest.

## Publisher’s Note

All claims expressed in this article are solely those of the authors and do not necessarily represent those of their affiliated organizations, or those of the publisher, the editors and the reviewers. Any product that may be evaluated in this article, or claim that may be made by its manufacturer, is not guaranteed or endorsed by the publisher.
