# Supplementary material for: ‘Bouncing Back’ From Subclinical Malaria: Inflammation and Erythrocytosis After Resolution of P. falciparum Infection in Gambian Children
Source: Front Immunol. 2022 Jan 28;13:780525. doi: 10.3389/fimmu.2022.780525 (PMC8831762; doi:10.3389/fimmu.2022.780525)
Supplement: Supplementary file 1 [file DataSheet_1.docx]

**‘Bouncing back’ from subclinical malaria: inflammation and erythrocytosis after resolution of *P. falciparum* infection in Gambian children**

Jason P. Mooney^1#^; Sophia M. DonVito^1^; Maimuna Jahateh^2^; Haddy Bittaye^2^; Marianne Keith^1^; Lauren J. Galloway^3^; Mortala Ndow^2^; Aubrey J. Cunnington^4^; Umberto D'Alessandro^2^; Christian Bottomley^5^; Eleanor M. Riley^1^

**SUPPLEMENTAL INFORMATION**

**Figures:**

**FS1,** related to Figure 6. Additional inflammatory cytokine levels in plasma.

**FS2,** related to Figure S1. Elevated CRP in PCR- children within the cohort.

**Tables:**

**TS1,** related to Figure 6**.** Plasma from malaria-infected, symptomatic Gambian children was used to serve as control reference values in the Luminex assay.

**TS2,** related to Figure 6**.** Luminex assay ‘Lower Limit of Quantification’ (LLOQ).

**TS3,** related to Figure 1**.** Height and weight comparisons between t_1_ and t_2_.

**TS4,** related to Figure 1**.** Eight children were removed for further analysis that were positive by microscopy at t_1_, but negative by RDT and varATS PCR.

**TS5,** related to Figure 1**.** Comparison of RDT, microscopy and varATS results from t_1_ and t_2_.

**TS6,** related to Figure 2**.** Breakdown of cohort by village and malaria status.

**TS7,** related to Figure 6. Level of inflammatory proteins in uninfected outliers.

**Methods:**

*P. falciparum diagnostic PCR reaction conditions.*

**Figure S1:**


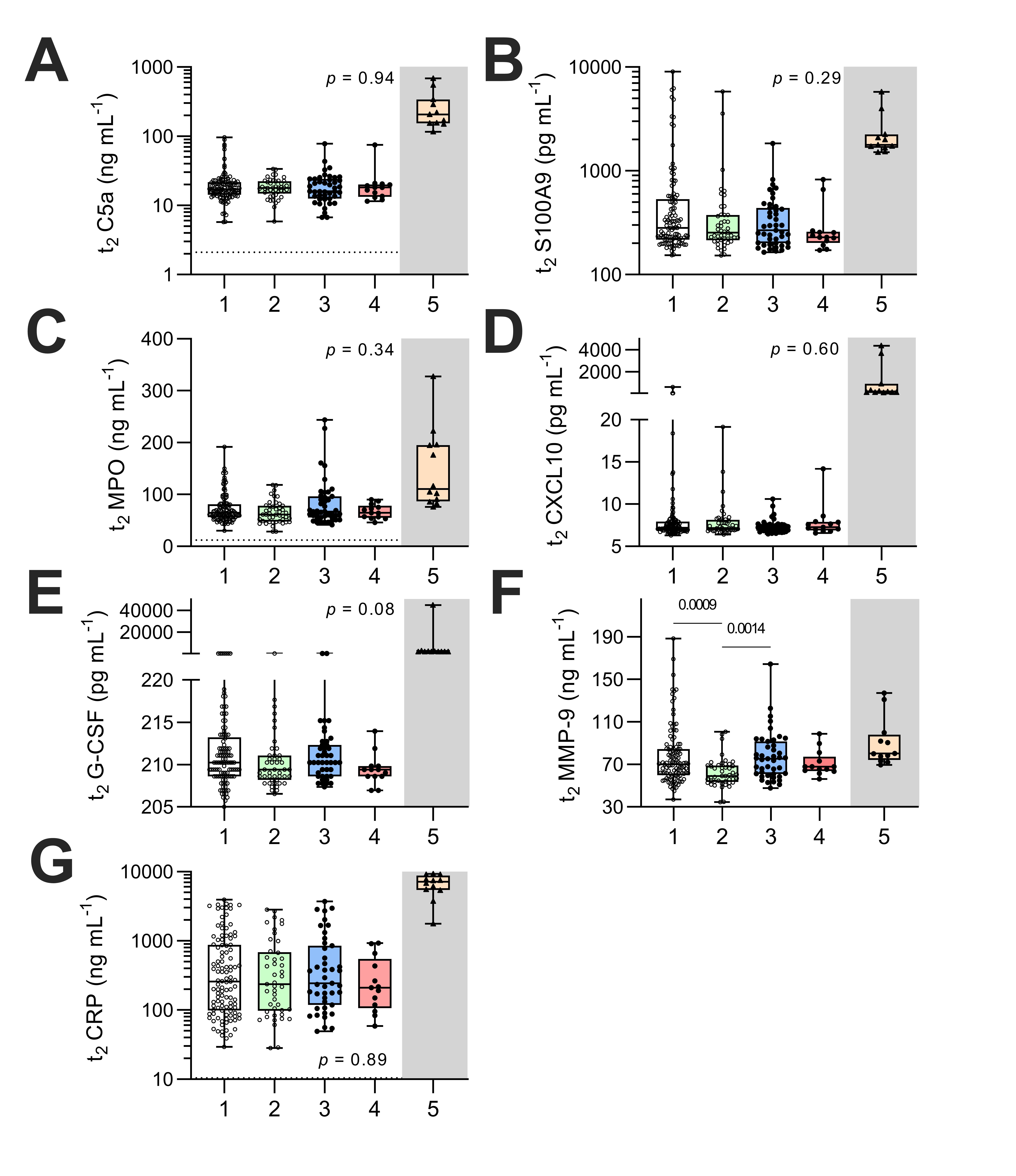


**Figure S1: Additional inflammatory cytokine levels in plasma, related to Figure 6.** Concentrations of soluble proteins in plasma measured by Luminex multiplex bead-based assay (Invitrogen) for **(A)** C5a (Complement component 5a), **(B)** S100a9 (S100 Calcium Binding Protein A9), **(C)** MPO (Myeloperoxidase), **(D)** CXCL10 (IP-10), **(E)** G-CSF (granulocyte-colony stimulating factor), **(F)** MMP-9 (Matrix metallopeptidase 9), and **(G)** C-reactive protein (CRP). Dotted lines represent ‘lower limit of quantification’ (LLOQ, **Table S2**). Data shown as box plots with min/max whiskers where dots represent each participant. Significant *p* values shown, calculated using a Kruskal-Wallis rank sum test followed by a post-hoc Dunn’s test with multiple comparisons. As a reference point (shown in grey box), protein concentrations for 12 Gambian children with acute, clinical malaria (Walther et al., 2009; Cunnington et al., 2013), with patient demographics shown in **Table S1**). Group IDs: #1 ‘controls’, #2 ‘anaemic’, #3 ‘resolved’, #4 ‘chronic’, & #5 ‘clinical malaria’.

**Figure S2:**


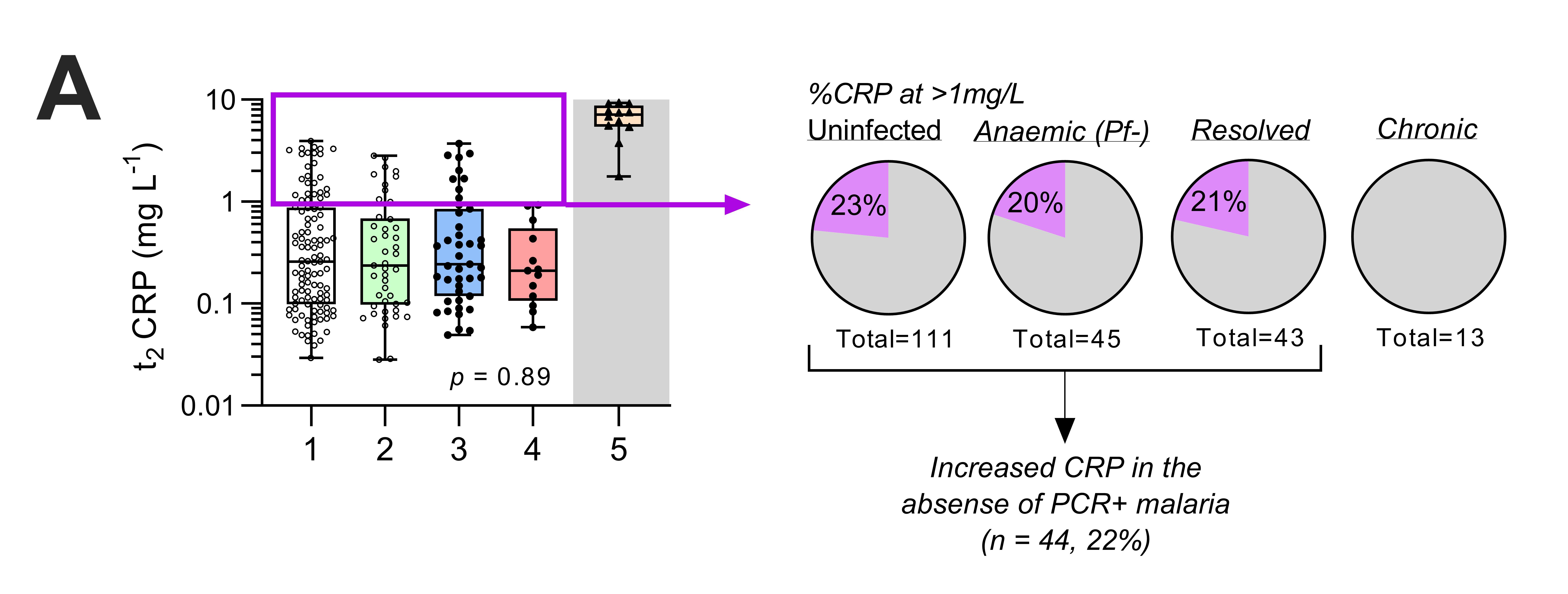


**FS2,** related to Figure S1. Elevated CRP in PCR- children within the cohort.

**(A)** The healthy pediatric range of CRP is less than 1 mg/L (Andropoulos, 2012). Within the PCR- children at recall, a total of 44 children showed elevated CRP (44/199, 22.1%). Group IDs: #1 ‘controls’, #2 ‘anaemic’, #3 ‘resolved’, #4 ‘chronic’, & #5 ‘clinical malaria’.

**Table S1:**

**Table S1,** related to Figure 6**.** Plasma from malaria-infected, symptomatic Gambian children was used to serve as control reference values in the Luminex assay. The data collected from these individuals is described in (Walther et al., 2009) and (Cunnington et al., 2013), with individual data shown below;

| ID | Date | Age (y) | Parasites/µL | Weight (kg) | Sex | Temp (°C) | Hb (g/dL) |
| --- | --- | --- | --- | --- | --- | --- | --- |
| 301 | 2008_09 | 11 | 208000 | 35 | Female | 39.0 | 10.6 |
| 307 | 2008_09 | 3 | 183375 | 12 | Male | 39.5 | 6.6 |
| 381 | 2008_09 | 11 | 230000 | 35 | Male | 36.4 | 13.4 |
| 476 | 2008_09 | 10 | 509000 | 20 | Male | 37.3 | 14.4 |
| 478 | 2008_09 | 5 | 3962 | 20 | Male | 38.3 | 8.1 |
| 485 | 2008_09 | 8 | 223890 | 22 | Male | 40.0 | 12.4 |
| 488 | 2008_09 | 6 | 597000 | 17 | Female | 37.6 | 9.3 |
| 492 | 2008_09 | 10 | 315700 | 27 | Female | 38.9 | 13.3 |
| 497 | 2008_09 | 5 | 394200 | 14 | Female | 39.5 | 11.8 |
| 505 | 2008_09 | 6 | 995300 | 16 | Female | 38.5 | 7.9 |
| 572 | 2009_10 | 4 | 591000 | 12 | Male | 37.7 | 12.3 |
| 589 | 2009_10 | 6 | 69800 | 14 | Female | 37.4 | 9.0 |

**Table S2:**

**Table S2,** related to Figure 6**.** For the Luminex assay of plasma, the ‘Upper Limit of Quantification’ (ULOQ) and ‘Lower Limit of Detection (LLOD) was calculated from manufacturer software. The ‘Lower Limit of Quantification (LLOQ)’ was determined by the LLOD multiplied by the dilution factor of the unknown plasma tested in the assay. *The LLOQ was then placed into graphs with the appropriate units.

| Luminex Kit IDs | | | *Assay Standard Values* | | | *Software Generated Assay Limits* | | *Assay Lower Limit Calculation* | | *LLOQ [units]* |
| --- | --- | --- | --- | --- | --- | --- | --- | --- | --- | --- |
| Analyte | Code# | Units | | Std1 | Std6 | ULOQ | LLOD | Plasma Dilution Factor (DF) | LLOQ (LLOD x DF)* | Line on Graph* |
| CRP | BR62 | pg/mL | | 27790 | 114.4 | 29031.7 | 103.8 | 100 | 10380.0 | 10.4 ng/mL |
| LBP | BR57 | pg/mL | | 164950 | 678.8 | 154713.7 | 699.6 | 100 | 69960.0 | 0.69 µg/mL |
| MPO | BR53 | pg/mL | | 27460 | 113.0 | 28367.5 | 121.5 | 100 | 12150.0 | 12.1 ng/mL |
| Ferritin | BR19 | pg/mL | | 6190 | 25.5 | 6213.0 | 24.3 | 100 | 2430.0 | 2.4 ng/mL |
| MMP-9 | BR14 | pg/mL | | 28460 | 117.1 | 28457.0 | 119.0 | 100 | 11900.0 | 11.9 ng/mL |
| TFR | BR13 | pg/mL | | 1335000 | 5493.8 | 134466.1 | 1329.5 | 100 | 132950.0 | 0.13 mg/mL |
| CD163 | BR28 | pg/mL | | 1324400 | 5450.2 | 132876.5 | 14561.4 | 2 | 29122.8 | 29 ng/mL |
| GCSF | BR54 | pg/mL | | 5730 | 23.6 | 5733.2 | 22.9 | 2 | 45.8 | 45.8 pg/mL |
| IL-6 | BR13 | pg/mL | | 1150 | 4.7 | 1150.6 | 4.6 | 2 | 9.2 | 9.2 pg/mL |
| IL-10 | BR22 | pg/mL | | 1010 | 4.2 | 1010.1 | 4.1 | 2 | 8.2 | 8.2 pg/mL |
| S100a9 | BR46 | pg/mL | | 5750 | 23.7 | 5737.0 | 24.1 | 2 | 48.2 | 48.2 pg/mL |
| C5a | BR18 | pg/mL | | 255950 | 1053.3 | 255867.4 | 1060.2 | 2 | 2120.4 | 2.1 ng/mL |
| CXCL10 | BR21 | pg/mL | | 300 | 1.2 | 300.1 | 1.2 | 2 | 2.5 | 2.5 pg/mL |
| IFNɣ | BR29 | pg/mL | | 10690 | 44.0 | 10707.0 | 43.3 | 2 | 86.6 | 86.6 pg/mL |
| TNFα | BR12 | pg/mL | | 1930 | 7.9 | 1931.9 | 7.87 | 2 | 15.7 | 15.7 pg/mL |

**Table S3:**

**Table S3,** related to Figure 1**.** After comparing height and weight between t_1_ and t_2_, twelve children showed a gain with both weight and height, whilst 14 children showed a loss. This was based on a child having either (1) a negative height change (≥5cm) and a weight loss >15% (i.e. shrinking), or (2) a positive height change (≥10cm) and a weight gain greater than 20% (i.e. exceptional growth). Therefore, these children were removed from all downstream analysis. An additional 7 children did not have recorded height and weight data to make this comparison and thus were removed from the analysis. Total t_2_ children removed, *n*=33.

|  | | | t_1_ | | t_2_ | | Change (%) | Change (cm) |
| --- | --- | --- | --- | --- | --- | --- | --- | --- |
| # | t_1_ ID | t_2_ ID | Weight (kg) | Height (m) | Weight (kg) | Height (m) | Weight | Height |
| Gain | | | | | | | | |
| 1 | A0738 | B011 | 20 | 1.24 | 37.2 | 1.53 | 46% | 29 |
| 2 | A0768 | B016 | 22 | 1.29 | 33.5 | 1.42 | 34% | 13 |
| 3 | A0030 | B031 | 18.2 | 1.35 | 43.2 | 1.5 | 58% | 15 |
| 4 | A0399 | B061 | 22.8 | 1.22 | 46.5 | 1.61 | 51% | 39 |
| 5 | A0791 | B073 | 20.9 | 1.21 | 29.9 | 1.46 | 30% | 25 |
| 6 | A0774 | B085 | 30.6 | 1.35 | 51.3 | 1.6 | 40% | 25 |
| 7 | A0776 | B086 | 27.7 | 1.3 | 41.1 | 1.54 | 33% | 24 |
| 8 | A0088 | B146 | 18.5 | 1.24 | 30.7 | 1.41 | 40% | 17 |
| 9 | A0101 | B149 | 36.2 | 1.51 | 62.3 | 1.7 | 42% | 19 |
| 10 | A0106 | B151 | 28.6 | 1.21 | 41.1 | 1.52 | 30% | 31 |
| 11 | A0759 | B172 | 28.4 | 1.36 | 35.8 | 1.55 | 21% | 19 |
| 12 | A1089 | B186 | 24.2 | 1.25 | 32.9 | 1.4 | 26% | 15 |
|  |  |  |  |  |  | **MEAN** | **38%** | **23** |
| Loss | | | | | | | | |
| 1 | A0732 | B003 | 31.6 | 1.46 | 22 | 1.25 | -44% | -21 |
| 2 | A0734 | B005 | 33.6 | 1.43 | 18.5 | 1.25 | -82% | -18 |
| 3 | A0742 | B008 | 47.2 | 1.64 | 20.4 | 1.22 | -131% | -42 |
| 4 | A0758 | B013 | 24.4 | 1.31 | 20.3 | 1.24 | -20% | -7 |
| 5 | A0772 | B017 | 24.1 | 1.28 | 18.7 | 1.23 | -29% | -5 |
| 6 | A0002 | B029 | 68 | 1.8 | 26.3 | 1.36 | -159% | -44 |
| 7 | A0736 | B083 | 41.4 | 1.55 | 25.5 | 1.31 | -62% | -24 |
| 8 | A0750 | B084 | 38.5 | 1.55 | 30.4 | 1.42 | -27% | -13 |
| 9 | A0111 | B153 | 37.1 | 1.66 | 30.9 | 1.45 | -20% | -21 |
| 10 | A0176 | B156 | 43.4 | 1.55 | 19.5 | 1.24 | -123% | -31 |
| 11 | A0175 | B162 | 40.3 | 1.55 | 28.4 | 1.38 | -42% | -17 |
| 12 | A0127 | B169 | 32.3 | 1.38 | 24.8 | 1.25 | -30% | -13 |
| 13 | A1087 | B185 | 29.4 | 1.45 | 22.2 | 1.23 | -32% | -22 |
| 14 | A1105 | B190 | 53.9 | 1.61 | 27.5 | 1.35 | -96% | -26 |
|  |  |  |  |  |  | **MEAN** | **-64%** | **-22** |

**Table S4:**

**Table S4,** related to Figure 1**.** Eight children were removed for further analysis that were positive by microscopy at t_1_, but negative by RDT and varATS PCR. *(1=positive, 0=negative)*

| *(Removed, n=8)* | | t_1_ | | | t_2_ | |
| --- | --- | --- | --- | --- | --- | --- |
| t_1_ ID | t_2_ ID | RDT | Microscopy | varATS | RDT | varATS |
| A0511 | B100 | 0 | 1 | 0 | 0 | 0 |
| A0560 | B107 | 0 | 1 | 0 | 0 | 0 |
| A0559 | B123 | 0 | 1 | 0 | 0 | 0 |
| A0040 | B141 | 0 | 1 | 0 | 0 | 0 |
| A0141 | B157 | 0 | 1 | 0 | 0 | 0 |
| A1632 | B221 | 0 | 1 | 0 | 0 | 0 |
| A1622 | B243 | 0 | 1 | 0 | 0 | 1 |
| A0089 | B147 | 0 | 1 | 0 | 0 | 1 |

**Table S5:**

**Table S5,** related to Figure 1**.** Comparison of RDT, microscopy and varATS results from t_1_ and t_2_. To note, all RDT and microscopy positive samples came up positive by varATS PCR. *(1=positive, 0=negative)*

| **t1 ID** | **t2 ID** | t1 | | | | t2 | | |
| --- | --- | --- | --- | --- | --- | --- | --- | --- |
|  |  | **Hb (g/L)** | **RDT** | **Microscopy** | **varATS** | **RDT** | **varATS** | **Final Group** |
|  |  |  | 7 | 6 | 57 | 1 | 13 |  |
| A0733 | B004 | 11.7 | 0 | 0 | 0 | 0 | 0 | #1 'control' |
| A0749 | B009 | 12.3 | 0 | 0 | 0 | 0 | 0 | #1 'control' |
| A0726 | B010 | 11.6 | 0 | 0 | 0 | 0 | 0 | #1 'control' |
| A0765 | B014 | 11.7 | 0 | 0 | 0 | 0 | 0 | #1 'control' |
| A0773 | B018 | 13 | 0 | 0 | 0 | 0 | 0 | #1 'control' |
| A0747 | B020 | 11.8 | 0 | 0 | 0 | 0 | 0 | #1 'control' |
| A0490 | B023 | 12.1 | 0 | 0 | 0 | 0 | 0 | #1 'control' |
| A0504 | B026 | 11.8 | 0 | 0 | 0 | 0 | 0 | #1 'control' |
| A0451 | B032 | 11.9 | 0 | 0 | 0 | 0 | 0 | #1 'control' |
| A0459 | B034 | 12.8 | 0 | 0 | 0 | 0 | 0 | #1 'control' |
| A0461 | B036 | 11.8 | 0 | 0 | 0 | 0 | 0 | #1 'control' |
| A0485 | B041 | 12.2 | 0 | 0 | 0 | 0 | 0 | #1 'control' |
| A0391 | B049 | 11.9 | 0 | 0 | 0 | 0 | 0 | #1 'control' |
| A0392 | B050 | 12.4 | 0 | 0 | 0 | 0 | 0 | #1 'control' |
| A0426 | B052 | 12.6 | 0 | 0 | 0 | 0 | 0 | #1 'control' |
| A0413 | B058 | 12.1 | 0 | 0 | 0 | 0 | 0 | #1 'control' |
| A0410 | B063 | 12.2 | 0 | 0 | 0 | 0 | 0 | #1 'control' |
| A0785 | B070 | 11.6 | 0 | 0 | 0 | 0 | 0 | #1 'control' |
| A0790 | B072 | 11.9 | 0 | 0 | 0 | 0 | 0 | #1 'control' |
| A0824 | B077 | 13.1 | 0 | 0 | 0 | 0 | 0 | #1 'control' |
| A0812 | B087 | 11.8 | 0 | 0 | 0 | 0 | 0 | #1 'control' |
| A0877 | B092 | 12.6 | 0 | 0 | 0 | 0 | 0 | #1 'control' |
| A0879 | B093 | 12 | 0 | 0 | 0 | 0 | 0 | #1 'control' |
| A0899 | B094 | 12.3 | 0 | 0 | 0 | 0 | 0 | #1 'control' |
| A0900 | B095 | 12 | 0 | 0 | 0 | 0 | 0 | #1 'control' |
| A0905 | B096 | 12.4 | 0 | 0 | 0 | 0 | 0 | #1 'control' |
| A0515 | B101 | 13.2 | 0 | 0 | 0 | 0 | 0 | #1 'control' |
| A0548 | B105 | 12.7 | 0 | 0 | 0 | 0 | 0 | #1 'control' |
| A0902 | B109 | 13 | 0 | 0 | 0 | 0 | 0 | #1 'control' |
| A0916 | B112 | 12.3 | 0 | 0 | 0 | 0 | 0 | #1 'control' |
| A0538 | B118 | 11.8 | 0 | 0 | 0 | 0 | 0 | #1 'control' |
| A0556 | B121 | 12.1 | 0 | 0 | 0 | 0 | 0 | #1 'control' |
| A0557 | B122 | 14.1 | 0 | 0 | 0 | 0 | 0 | #1 'control' |
| A0315 | B129 | 13 | 0 | 0 | 0 | 0 | 0 | #1 'control' |
| A0337 | B130 | 12.6 | 0 | 0 | 0 | 0 | 0 | #1 'control' |
| A0351 | B131 | 12.6 | 0 | 0 | 0 | 0 | 0 | #1 'control' |
| A0354 | B134 | 14.3 | 0 | 0 | 0 | 0 | 0 | #1 'control' |
| A0355 | B135 | 12.7 | 0 | 0 | 0 | 0 | 0 | #1 'control' |
| A0356 | B136 | 13.1 | 0 | 0 | 0 | 0 | 0 | #1 'control' |
| A0361 | B139 | 13.5 | 0 | 0 | 0 | 0 | 0 | #1 'control' |
| A0044 | B142 | 11.9 | 0 | 0 | 0 | 0 | 0 | #1 'control' |
| A0046 | B143 | 11.6 | 0 | 0 | 0 | 0 | 0 | #1 'control' |
| A0058 | B144 | 12.2 | 0 | 0 | 0 | 0 | 0 | #1 'control' |
| A0917 | B168 | 12.8 | 0 | 0 | 0 | 0 | 0 | #1 'control' |
| A0976 | B173 | 12.1 | 0 | 0 | 0 | 0 | 0 | #1 'control' |
| A1014 | B176 | 12.8 | 0 | 0 | 0 | 0 | 0 | #1 'control' |
| A1024 | B177 | 12.7 | 0 | 0 | 0 | 0 | 0 | #1 'control' |
| A1056 | B178 | 12.5 | 0 | 0 | 0 | 0 | 0 | #1 'control' |
| A1058 | B179 | 12.3 | 0 | 0 | 0 | 0 | 0 | #1 'control' |
| A1076 | B181 | 11.6 | 0 | 0 | 0 | 0 | 0 | #1 'control' |
| A1094 | B187 | 13.6 | 0 | 0 | 0 | 0 | 0 | #1 'control' |
| A1040 | B192 | 12.3 | 0 | 0 | 0 | 0 | 0 | #1 'control' |
| A1180 | B193 | 12.8 | 0 | 0 | 0 | 0 | 0 | #1 'control' |
| A1181 | B194 | 13.1 | 0 | 0 | 0 | 0 | 0 | #1 'control' |
| A1208 | B197 | 12.3 | 0 | 0 | 0 | 0 | 0 | #1 'control' |
| A1236 | B200 | 11.9 | 0 | 0 | 0 | 0 | 0 | #1 'control' |
| A1267 | B202 | 12.1 | 0 | 0 | 0 | 0 | 0 | #1 'control' |
| A1373 | B213 | 12.3 | 0 | 0 | 0 | 0 | 0 | #1 'control' |
| A1384 | B214 | 12.4 | 0 | 0 | 0 | 0 | 0 | #1 'control' |
| A1627 | B219 | 13.4 | 0 | 0 | 0 | 0 | 0 | #1 'control' |
| A1640 | B223 | 12.7 | 0 | 0 | 0 | 0 | 0 | #1 'control' |
| A1648 | B226 | 11.7 | 0 | 0 | 0 | 0 | 0 | #1 'control' |
| A1512 | B227 | 12.2 | 0 | 0 | 0 | 0 | 0 | #1 'control' |
| A1513 | B228 | 11.9 | 0 | 0 | 0 | 0 | 0 | #1 'control' |
| A1534 | B233 | 13.9 | 0 | 0 | 0 | 0 | 0 | #1 'control' |
| A1558 | B239 | 13.6 | 0 | 0 | 0 | 0 | 0 | #1 'control' |
| A1573 | B242 | 11.6 | 0 | 0 | 0 | 0 | 0 | #1 'control' |
| A1588 | B246 | 12.9 | 0 | 0 | 0 | 0 | 0 | #1 'control' |
| A1599 | B249 | 12.7 | 0 | 0 | 0 | 0 | 0 | #1 'control' |
| A1531 | B251 | 13.1 | 0 | 0 | 0 | 0 | 0 | #1 'control' |
| A1594 | B256 | 13.4 | 0 | 0 | 0 | 0 | 0 | #1 'control' |
| A1611 | B258 | 12.9 | 0 | 0 | 0 | 0 | 0 | #1 'control' |
| A1612 | B259 | 11.9 | 0 | 0 | 0 | 0 | 0 | #1 'control' |
| A1619 | B261 | 12.7 | 0 | 0 | 0 | 0 | 0 | #1 'control' |
| A1624 | B262 | 12.5 | 0 | 0 | 0 | 0 | 0 | #1 'control' |
| A0739 | B007 | 11.5 | 0 | 0 | 0 | 0 | 0 | #2 'anaemic' |
| A0757 | B012 | 8.7 | 0 | 0 | 0 | 0 | 0 | #2 'anaemic' |
| A0744 | B015 | 10.6 | 0 | 0 | 0 | 0 | 0 | #2 'anaemic' |
| A0782 | B019 | 10.7 | 0 | 0 | 0 | 0 | 0 | #2 'anaemic' |
| A0375 | B045 | 10.3 | 0 | 0 | 0 | 0 | 0 | #2 'anaemic' |
| A0404 | B056 | 11.3 | 0 | 0 | 0 | 0 | 0 | #2 'anaemic' |
| A0761 | B068 | 10.9 | 0 | 0 | 0 | 0 | 0 | #2 'anaemic' |
| A0763 | B069 | 11.1 | 0 | 0 | 0 | 0 | 0 | #2 'anaemic' |
| A0832 | B076 | 9.9 | 0 | 0 | 0 | 0 | 0 | #2 'anaemic' |
| A0876 | B091 | 10.3 | 0 | 0 | 0 | 0 | 0 | #2 'anaemic' |
| A0508 | B099 | 9.9 | 0 | 0 | 0 | 0 | 0 | #2 'anaemic' |
| A0539 | B103 | 10.6 | 0 | 0 | 0 | 0 | 0 | #2 'anaemic' |
| A0892 | B108 | 5.5 | 0 | 0 | 0 | 0 | 0 | #2 'anaemic' |
| A0906 | B110 | 9.5 | 0 | 0 | 0 | 0 | 0 | #2 'anaemic' |
| A0915 | B111 | 10.9 | 0 | 0 | 0 | 0 | 0 | #2 'anaemic' |
| A0522 | B115 | 10.5 | 0 | 0 | 0 | 0 | 0 | #2 'anaemic' |
| A0528 | B117 | 11.5 | 0 | 0 | 0 | 0 | 0 | #2 'anaemic' |
| A0547 | B120 | 11.3 | 0 | 0 | 0 | 0 | 0 | #2 'anaemic' |
| A0565 | B124 | 10.3 | 0 | 0 | 0 | 0 | 0 | #2 'anaemic' |
| A0276 | B127 | 11.2 | 0 | 0 | 0 | 0 | 0 | #2 'anaemic' |
| A0311 | B128 | 11.3 | 0 | 0 | 0 | 0 | 0 | #2 'anaemic' |
| A0369 | B140 | 11.4 | 0 | 0 | 0 | 0 | 0 | #2 'anaemic' |
| A0070 | B145 | 11.5 | 0 | 0 | 0 | 0 | 0 | #2 'anaemic' |
| A0091 | B148 | 11.2 | 0 | 0 | 0 | 0 | 0 | #2 'anaemic' |
| A0110 | B152 | 10.4 | 0 | 0 | 0 | 0 | 0 | #2 'anaemic' |
| A0118 | B154 | 10 | 0 | 0 | 0 | 0 | 0 | #2 'anaemic' |
| A0124 | B155 | 10.9 | 0 | 0 | 0 | 0 | 0 | #2 'anaemic' |
| A0434 | B163 | 10.8 | 0 | 0 | 0 | 0 | 0 | #2 'anaemic' |
| A0450 | B167 | 11.5 | 0 | 0 | 0 | 0 | 0 | #2 'anaemic' |
| A0990 | B174 | 11.2 | 0 | 0 | 0 | 0 | 0 | #2 'anaemic' |
| A1000 | B175 | 8.5 | 0 | 0 | 0 | 0 | 0 | #2 'anaemic' |
| A1072 | B180 | 9.9 | 0 | 0 | 0 | 0 | 0 | #2 'anaemic' |
| A1078 | B182 | 8.9 | 0 | 0 | 0 | 0 | 0 | #2 'anaemic' |
| A1083 | B183 | 10.1 | 0 | 0 | 0 | 0 | 0 | #2 'anaemic' |
| A1086 | B184 | 9.9 | 0 | 0 | 0 | 0 | 0 | #2 'anaemic' |
| A1098 | B188 | 9.3 | 0 | 0 | 0 | 0 | 0 | #2 'anaemic' |
| A1100 | B189 | 10.7 | 0 | 0 | 0 | 0 | 0 | #2 'anaemic' |
| A1055 | B191 | 9.6 | 0 | 0 | 0 | 0 | 0 | #2 'anaemic' |
| A1195 | B195 | 10.2 | 0 | 0 | 0 | 0 | 0 | #2 'anaemic' |
| A1205 | B196 | 10.2 | 0 | 0 | 0 | 0 | 0 | #2 'anaemic' |
| A1213 | B199 | 9.3 | 0 | 0 | 0 | 0 | 0 | #2 'anaemic' |
| A1257 | B201 | 10.9 | 0 | 0 | 0 | 0 | 0 | #2 'anaemic' |
| A1290 | B203 | 11.3 | 0 | 0 | 0 | 0 | 0 | #2 'anaemic' |
| A1294 | B204 | 6.1 | 0 | 0 | 0 | 0 | 0 | #2 'anaemic' |
| A1299 | B205 | 10 | 0 | 0 | 0 | 0 | 0 | #2 'anaemic' |
| A1303 | B206 | 10.6 | 0 | 0 | 0 | 0 | 0 | #2 'anaemic' |
| A1289 | B207 | 10.8 | 0 | 0 | 0 | 0 | 0 | #2 'anaemic' |
| A1335 | B208 | 11.5 | 0 | 0 | 0 | 0 | 0 | #2 'anaemic' |
| A1340 | B209 | 11.5 | 0 | 0 | 0 | 0 | 0 | #2 'anaemic' |
| A1353 | B210 | 9.3 | 0 | 0 | 0 | 0 | 0 | #2 'anaemic' |
| A1359 | B211 | 11.2 | 0 | 0 | 0 | 0 | 0 | #2 'anaemic' |
| A1369 | B212 | 8.1 | 0 | 0 | 0 | 0 | 0 | #2 'anaemic' |
| A1465 | B215 | 10 | 0 | 0 | 0 | 0 | 0 | #2 'anaemic' |
| A1467 | B216 | 10.8 | 0 | 0 | 0 | 0 | 0 | #2 'anaemic' |
| A1621 | B217 | 10.4 | 0 | 0 | 0 | 0 | 0 | #2 'anaemic' |
| A1626 | B218 | 9.9 | 0 | 0 | 0 | 0 | 0 | #2 'anaemic' |
| A1629 | B220 | 10.7 | 0 | 0 | 0 | 0 | 0 | #2 'anaemic' |
| A1633 | B222 | 11 | 0 | 0 | 0 | 0 | 0 | #2 'anaemic' |
| A1647 | B225 | 9.8 | 0 | 0 | 0 | 0 | 0 | #2 'anaemic' |
| A1522 | B230 | 10.4 | 0 | 0 | 0 | 0 | 0 | #2 'anaemic' |
| A1527 | B231 | 11.5 | 0 | 0 | 0 | 0 | 0 | #2 'anaemic' |
| A1528 | B232 | 7.1 | 0 | 0 | 0 | 0 | 0 | #2 'anaemic' |
| A1537 | B234 | 10.3 | 0 | 0 | 0 | 0 | 0 | #2 'anaemic' |
| A1540 | B236 | 10.4 | 0 | 0 | 0 | 0 | 0 | #2 'anaemic' |
| A1555 | B237 | 11.4 | 0 | 0 | 0 | 0 | 0 | #2 'anaemic' |
| A1566 | B240 | 9.4 | 0 | 0 | 0 | 0 | 0 | #2 'anaemic' |
| A1568 | B241 | 10.5 | 0 | 0 | 0 | 0 | 0 | #2 'anaemic' |
| A1583 | B244 | 10.5 | 0 | 0 | 0 | 0 | 0 | #2 'anaemic' |
| A1586 | B245 | 10.3 | 0 | 0 | 0 | 0 | 0 | #2 'anaemic' |
| A1592 | B247 | 9.9 | 0 | 0 | 0 | 0 | 0 | #2 'anaemic' |
| A1596 | B248 | 11.5 | 0 | 0 | 0 | 0 | 0 | #2 'anaemic' |
| A1572 | B253 | 7.3 | 0 | 0 | 0 | 0 | 0 | #2 'anaemic' |
| A1575 | B254 | 10.7 | 0 | 0 | 0 | 0 | 0 | #2 'anaemic' |
| A1609 | B257 | 9.2 | 0 | 0 | 0 | 0 | 0 | #2 'anaemic' |
| A1618 | B260 | 10.7 | 0 | 0 | 0 | 0 | 0 | #2 'anaemic' |
| A1639 | B263 | 10.8 | 0 | 0 | 0 | 0 | 0 | #2 'anaemic' |
| A1650 | B264 | 10.1 | 0 | 0 | 0 | 0 | 0 | #2 'anaemic' |
| A0737 | B006 | 12.1 | 0 | 0 | 1 | 0 | 0 | #3 'resolved' |
| A0476 | B021 | 11.9 | 0 | 0 | 1 | 0 | 0 | #3 'resolved' |
| A0477 | B022 | 11.9 | 0 | 0 | 1 | 0 | 0 | #3 'resolved' |
| A0491 | B024 | 11.7 | 0 | 0 | 1 | 0 | 0 | #3 'resolved' |
| A0499 | B025 | 12.4 | 0 | 0 | 1 | 0 | 0 | #3 'resolved' |
| A0505 | B027 | 12.2 | 0 | 0 | 1 | 0 | 0 | #3 'resolved' |
| A0492 | B028 | 11.7 | 0 | 0 | 1 | 0 | 0 | #3 'resolved' |
| A0014 | B030 | 11.5 | 1 | 0 | 1 | 0 | 0 | #3 'resolved' |
| A0458 | B033 | 13.1 | 0 | 0 | 1 | 0 | 0 | #3 'resolved' |
| A0460 | B035 | 12 | 0 | 0 | 1 | 0 | 0 | #3 'resolved' |
| A0467 | B037 | 11.4 | 0 | 0 | 1 | 0 | 0 | #3 'resolved' |
| A0471 | B038 | 12.9 | 0 | 0 | 1 | 0 | 0 | #3 'resolved' |
| A0481 | B039 | 12.4 | 0 | 1 | 1 | 0 | 0 | #3 'resolved' |
| A0483 | B040 | 10.9 | 0 | 0 | 1 | 0 | 0 | #3 'resolved' |
| A0488 | B042 | 11 | 0 | 0 | 1 | 0 | 0 | #3 'resolved' |
| A0489 | B043 | 11 | 0 | 0 | 1 | 0 | 0 | #3 'resolved' |
| A0502 | B044 | 11.1 | 0 | 1 | 1 | 0 | 0 | #3 'resolved' |
| A0382 | B046 | 14.6 | 0 | 0 | 1 | 0 | 0 | #3 'resolved' |
| A0383 | B047 | 13.6 | 0 | 0 | 1 | 0 | 0 | #3 'resolved' |
| A0386 | B048 | 12.3 | 0 | 0 | 1 | 0 | 0 | #3 'resolved' |
| A0396 | B051 | 10.1 | 0 | 0 | 1 | 0 | 0 | #3 'resolved' |
| A0431 | B053 | 14.1 | 0 | 0 | 1 | 0 | 0 | #3 'resolved' |
| A0430 | B054 | 13.1 | 0 | 0 | 1 | 0 | 0 | #3 'resolved' |
| A0418 | B055 | 11.3 | 0 | 0 | 1 | 0 | 0 | #3 'resolved' |
| A0384 | B057 | 12.4 | 0 | 0 | 1 | 0 | 0 | #3 'resolved' |
| A0395 | B059 | 10.9 | 0 | 0 | 1 | 0 | 0 | #3 'resolved' |
| A0397 | B060 | 13.5 | 0 | 0 | 1 | 0 | 0 | #3 'resolved' |
| A0408 | B062 | 11.8 | 0 | 0 | 1 | 0 | 0 | #3 'resolved' |
| A0417 | B064 | 12.9 | 0 | 0 | 1 | 0 | 0 | #3 'resolved' |
| A0419 | B065 | 12.4 | 0 | 0 | 1 | 0 | 0 | #3 'resolved' |
| A0432 | B066 | 11.2 | 0 | 0 | 1 | 0 | 0 | #3 'resolved' |
| A0420 | B067 | 11.9 | 0 | 0 | 1 | 0 | 0 | #3 'resolved' |
| A0836 | B080 | 11.7 | 0 | 0 | 1 | 0 | 0 | #3 'resolved' |
| A0875 | B090 | 11.8 | 1 | 1 | 1 | 0 | 0 | #3 'resolved' |
| A0506 | B097 | 10.3 | 0 | 0 | 1 | 0 | 0 | #3 'resolved' |
| A0507 | B098 | 12.5 | 0 | 0 | 1 | 0 | 0 | #3 'resolved' |
| A0520 | B102 | 13.3 | 0 | 0 | 1 | 0 | 0 | #3 'resolved' |
| A0542 | B104 | 12.3 | 0 | 0 | 1 | 0 | 0 | #3 'resolved' |
| A0552 | B106 | 11.5 | 0 | 0 | 1 | 0 | 0 | #3 'resolved' |
| A0525 | B116 | 12.1 | 0 | 0 | 1 | 0 | 0 | #3 'resolved' |
| A0540 | B119 | 11.1 | 0 | 0 | 1 | 0 | 0 | #3 'resolved' |
| A0441 | B164 | 11.6 | 0 | 0 | 1 | 0 | 0 | #3 'resolved' |
| A0445 | B166 | 13.5 | 0 | 0 | 1 | 0 | 0 | #3 'resolved' |
| A1557 | B238 | 10.7 | 0 | 0 | 1 | 0 | 0 | #3 'resolved' |
| A0725 | B001 | 11.5 | 1 | 0 | 1 | 0 | 1 | #4 'chronic' |
| A0728 | B002 | 9.8 | 1 | 1 | 1 | 0 | 1 | #4 'chronic' |
| A0858 | B081 | 11.7 | 0 | 0 | 1 | 0 | 1 | #4 'chronic' |
| A0754 | B082 | 11.8 | 0 | 0 | 1 | 0 | 1 | #4 'chronic' |
| A0844 | B089 | 9.2 | 0 | 0 | 1 | 0 | 1 | #4 'chronic' |
| A0920 | B113 | 11.5 | 1 | 1 | 1 | 0 | 1 | #4 'chronic' |
| A0519 | B114 | 13.6 | 0 | 0 | 1 | 0 | 1 | #4 'chronic' |
| A0352 | B132 | 12.3 | 1 | 0 | 1 | 0 | 1 | #4 'chronic' |
| A0353 | B133 | 12.7 | 0 | 0 | 1 | 0 | 1 | #4 'chronic' |
| A0358 | B138 | 11.8 | 0 | 0 | 1 | 0 | 1 | #4 'chronic' |
| A0105 | B150 | 11.8 | 1 | 0 | 1 | 0 | 1 | #4 'chronic' |
| A0442 | B165 | 14 | 0 | 0 | 1 | 0 | 1 | #4 'chronic' |
| A0748 | B171 | 10.4 | 0 | 1 | 1 | 1 | 1 | #4 'chronic' |

**Table S6:**

**Table S6,** related to Figure 2**.** Breakdown of cohort by village and malaria status.

| Cluster | # | Village Name | #1 Uninfected (n) | #2 Anaemic | #3 Resolved | #4 Chronic |
| --- | --- | --- | --- | --- | --- | --- |
| i | 1 | Koro Jula Kunda | 0 | 3 | 0 | 0 |
|  | 2 | Koro Numu Kunda | 0 | 1 | 0 | 1 |
|  | 3 | Sare Talata | 1 | 0 | 0 | 0 |
|  | 4 | Busura Alieu | 2 | 0 | 0 | 0 |
| ii | 5 | Sare Dembel Jawo | 0 | 1 | 0 | 0 |
|  | 6 | Kosemari | 0 | 1 | 0 | 0 |
|  | 7 | Sare Mamudu | 5 | 0 | 0 | 3 |
|  | 8 | Hella Kunda | 2 | 1 | 0 | 0 |
| iii | 9 | Sotuma Sere | 5 | 4 | 17 | 1 |
| iv | 10 | Numuyel | 6 | 0 | 15 | 0 |
|  | 11 | Gambisara | 0 | 0 | 1 | 0 |
| v | 12 | Tamba Sansang | 5 | 6 | 7 | 1 |
| vi | 13 | Kundam MaFatty | 8 | 4 | 1 | 1 |
| vii | 14 | Dandugu | 0 | 0 | 0 | 1 |
|  | 15 | Sanunding | 10 | 7 | 2 | 4 |
|  | 16 | Kulinto | 0 | 0 | 0 | 1 |
| viii | 17 | Dingiri | 8 | 9 | 0 | 0 |
| ix | 18 | Sinchang Jabo | 0 | 1 | 0 | 0 |
|  | 19 | Suduwol | 5 | 9 | 0 | 0 |
| x | 20 | Garawoll | 2 | 7 | 0 | 0 |
| xi | 21 | N'yamanari | 16 | 23 | 1 | 0 |

**Table S7:**

**TS7,** related to Figure 6. Level of inflammatory proteins in uninfected outliers. Outliers within the datasets were calculated by ROUT (robust regression and outlier removal) with Q = 1%. Next, the % of the uninfected group which fell above the mean or median of the other groups was calculated.

| Analysis of Uninfected ‘Control’ Group | | | Clinical, Acute Pf+ | % Uninfected Above Mean of **'Clinical'** group | Resolved | % Uninfected Above Median of **'Resolved'** group | Chronic | % Uninfected Above Median of **'Chronic'** group |
| --- | --- | --- | --- | --- | --- | --- | --- | --- |
| **Analyte** | **# Samples** | **# Outliers** | **Mean** | **%** | **Median** | **%** | **Median** | **%** |
| S100a9 | 156 | 26 | 2368 | 7% | 268 | 51% | 230 | 67% |
| CRP | 156 | 26 | 6627 | - | 245 | 51% | 211 | 54% |
| CXCL10 | 156 | 15 | 923 | - | 7 | 49% | 7 | 47% |
| MMP9 | 156 | 11 | 90 | 15% | 75 | 30% | 68 | 47% |
| CD163 | 156 | 9 | 855 | 8% | 253 | 52% | 587 | 21% |
| C5a | 156 | 8 | 276 | - | 16 | 64% | 18 | 44% |
| MPO | 156 | 8 | 148 | 1% | 67 | 44% | 65 | 49% |
| GCSF | 155 | 8 | 6046 | - | 210 | 43% | 209 | 53% |
| IFNg | 156 | 5 | 1935 | - | 98 | 17% | 92 | 38% |
| IL6 | 156 | 5 | 2980 | - | 8 | 31% | 7 | 43% |
| IL10 | 156 | 3 | 2763 | - | 9 | 47% | 11 | 19% |
| TNFa | 156 | 2 | 114 | - | 9 | 24% | 9 | 24% |

**SUPPLEMENTAL MATERIALS AND METHODS**

*P. falciparum rtPCR and qPCR*

Three x 3-mm dried blood spots were punched into 96 deep-well plates, digested in 20 µl of proteinase K and 180 µL of ATL buffer solution and extracted using the QIAamp 96 DNA QIAcube HT Kit (Qiagen). Extracted DNA was stored at -70˚C and defrosted at 4˚C prior to use.

*varATS PCR reaction conditions*

Based on methods described previously ((Hofmann et al., 2015)), samples and standards were run in duplicate on Roche LightCycler 480 II in 384-well plates in total reaction volumes of 12 μL (9 μL mastermix (0.5μL PCR-grade H2O, 1.0 μL (0.8μM), varATS forward primer (5’–CCCATACACAACCAAYTGGA), 1.0 μL (0.8μM) varATS reverse primer (5’–TTCGCACATATCTCTATGTCTAT), 0.5 μL (0.4μM) varATS probe (5’–[6FAM]TRTTCCATAAATGGT[BHQ1] and 6.0 μL (1x) Agilent Brilliant III Ultra-Fast qPCR Master Mix, Cat. #600880)) and 3 μL of sample or standard (NIBSC code 04/176 (Padley et al., 2008) in 10-fold dilutions to give final concentrations of 1x10^8^ to 1x10^1^ IU/mL, equivalent to 4.7x10^4^ to 4.7x10^-3^ parasites/μL (Hofmann et al., 2015)). Thermocycler conditions were pre-incubation at 50˚C for 2 minutes, denaturation at 95˚C for 10 minutes, 45 cycles of 15-seconds denaturation at 95˚C and 1-minute annealing and elongation at 55˚C with data acquisition, and a final cooling step at 40˚C for 30 seconds.

*18s PCR reaction conditions*

Based on methods described previously ((Pinheirob et al., 1993; Singh et al., 1999; Rougemont et al., 2004)), and based on institutional protocol established at the MRC molecular diagnostic unit in The Gambia (‘DETECTION OF PLASMODIUM INFECTION BY NESTED PCR ASSAY’, SOP-QUA-001v3.0). To summarise, samples were run in 96-well plates with controls including DNA from *P.f.* strain 3D7 as a positive control, uninfected blood, or no DNA negative controls. Samples were run in a 15 μL total reaction volume using the following conditions; 1.5 μL reaction buffer (Thermopol), 0.12 μL Taq DNA Polymerase (5 U/μL), 7.88μL ultrapure water, 0.3 μL dNTPs, 4μL extracted DNA template, 0.6μL of each primer at 0.4 μM (rPLU6 = 5’-TTAAAATTGTTGCAGTTAAAACG-3’, and rPLU5new = 5’-CYTGTTGTTGCCTTAAACTTC-3’). Thermocycler conditions were denaturation at 94˚C for 3 minutes, 24 cycles of 30 seconds denaturation at 94˚C and annealing and elongation at 58˚C for 30 seconds and 72˚C for 45 seconds, and a final step at 72˚C for 5 min. This resulted in a PCR product size of 1,200 bp. Next, a second ‘nested’ PCR reaction was performed. Here, samples were run with in 15μL total reaction volume using the following conditions; 1.5 μL reaction buffer (Thermopol), 0.12 μL Taq DNA Polymerase (5 U/μL), 11.18 μL ultrapure water, 1 μL PCR product from the previous reaction (‘nest 1’), 0.45 μL of each primer at 0.3 μM (rFAL1 – 5’-TTAAACTGGTTTGGGAAAACCAAATATATT-3’, and rFAL2 – 5’=ACACAATAGACTCAATCATGACTACCCGTC-3’). Thermocycler conditions were denaturation at 94˚C for 3 minutes, 29 cycles of 30 seconds denaturation at 94˚C and annealing and elongation at 60˚C for 30 seconds and 72˚C for 45 seconds, and a final step at 72˚C for 5 min. This resulted in a PCR product size of 205bp. PCR products were visualised after running on a QIAxcel (Qiagen), based on manufacturer instructions.

Andropoulos, D.B. (2012). "Appendix B: Pediatric Normal Laboratory Values," in *Gregory's Pediatric Anesthesia*.), 1300-1314.

Cunnington, A.J., Bretscher, M.T., Nogaro, S.I., Riley, E.M., and Walther, M. (2013). Comparison of parasite sequestration in uncomplicated and severe childhood Plasmodium falciparum malaria. *Journal of Infection* 67(3)**,** 220-230. doi: <https://doi.org/10.1016/j.jinf.2013.04.013>.

Hofmann, N., Mwingira, F., Shekalaghe, S., Robinson, L.J., Mueller, I., and Felger, I. (2015). Ultra-Sensitive Detection of Plasmodium falciparum by Amplification of Multi-Copy Subtelomeric Targets. *PLOS Medicine* 12(3)**,** e1001788. doi: 10.1371/journal.pmed.1001788.

Padley, D.J., Heath, A.B., Sutherland, C., Chiodini, P.L., Baylis, S.A., and the Collaborative Study, G. (2008). Establishment of the 1st World Health Organization International Standard for Plasmodium falciparum DNA for nucleic acid amplification technique (NAT)-based assays. *Malaria Journal* 7(1)**,** 139. doi: 10.1186/1475-2875-7-139.

Pinheirob, V.E., Thaithongc, S., and Browna, K.N. (1993). High sensitivity of detection of human malaria parasites by the use of nested polymerase chain reaction. *Mol. Biochem. Parasitol* 61**,** 315-320.

Rougemont, M., Van Saanen, M., Sahli, R., Hinrikson, H.P., Bille, J., and Jaton, K. (2004). Detection of four Plasmodium species in blood from humans by 18S rRNA gene subunit-based and species-specific real-time PCR assays. *Journal of clinical microbiology* 42(12)**,** 5636-5643.

Singh, B., Bobogare, A., Cox-Singh, J., Snounou, G., Abdullah, M.S., and Rahman, H.A. (1999). A genus- and species-specific nested polymerase chain reaction malaria detection assay for epidemiologic studies. *Am J Trop Med Hyg* 60(4)**,** 687-692. doi: 10.4269/ajtmh.1999.60.687.

Walther, M., Jeffries, D., Finney, O.C., Njie, M., Ebonyi, A., Deininger, S., et al. (2009). Distinct Roles for FOXP3+ and FOXP3− CD4+ T Cells in Regulating Cellular Immunity to Uncomplicated and Severe Plasmodium falciparum Malaria. *PLOS Pathogens* 5(4)**,** e1000364. doi: 10.1371/journal.ppat.1000364.
